# Psychiatric disorders after deep brain stimulation of the subthalamic nucleus in Parkinson’s disease: a systematic review

**DOI:** 10.31744/einstein_journal/2024RW0182

**Published:** 2024-07-02

**Authors:** Caio de Almeida Lellis, Marco Alejandro Menacho Herbas, Ledismar José da Silva

**Affiliations:** 1 Escola de Ciências Médicas e da Vida Pontifícia Universidade Católica de Goiás Goiânia GO Brazil Escola de Ciências Médicas e da Vida, Pontifícia Universidade Católica de Goiás, Goiânia, GO, Brazil.

**Keywords:** Parkinson’s disease, Deep brain stimulation, Subthalamic nucleus, Mental disorders, Social behavior disorders

## Abstract

**Objective:**

To evaluate the psychiatric alterations resulting from deep brain stimulation of the subthalamic nucleus in the management of Parkinson’s disease.

**Methods:**

Articles were searched using three databases: Public/Publisher MEDLINE, Virtual Health Library, and Cochrane Library.

**Results:**

Eleven studies were included in the analysis. Manic syndrome alone was reported in two of the 11 studies analyzed. Psychosis alone was not reported in any of them, but it was found in association with other psychiatric alterations in two studies, not including manic syndrome. In one case report, hypersexuality was associated with depression and self-alienation. Depressive disorder was the most frequent psychiatric disorder after deep brain stimulation of the subthalamic nucleus, according to five of the reviewed articles, encompassing 26 patients. In four of these articles, depression was associated with other psychiatric disorders, such as psychosis, suicidal ideation, hypersexuality, and anxiety. Hypomanic syndrome was reported in two cases.

**Conclusion:**

More common psychiatric disorders related to the neuroanatomy of the nucleus were observed, probably because of the microlesions caused by the implantation of deep brain stimulation and the regulation of the stimulation of the device. The most common disorders include depression, mania/hypomania, psychosis, anxiety, suicidal ideation, and hypersexuality.

## INTRODUCTION

Parkinson’s disease (PD), first described in 1817 by the surgeon James Parkinson, is a chronic and progressive neurodegenerative disorder that causes motor and non-motor symptoms. It is the second most common chronic neurodegenerative disease worldwide, surpassed only by Alzheimer’s disease.^(1)^

It belongs to a group of synucleinopathies, and its main characteristic is the accumulation of proteins, such as alpha-synuclein, in the nervous tissue. Consequently, there is a loss of dopaminergic neurons and the development of the cardinal symptoms of PD, such as rigidity, akinesia, bradykinesia, tremor, and postural instability.^1^

Surgical treatment for PD was proposed in 1947 when Spiegel and Wycis described a stereotaxic apparatus that provided subsidies for the location of brain structures.^(4)^ In the late 1980s, deep brain stimulation (DBS) of the subthalamic nucleus (STN) was proposed as a therapeutic alternative to ablation surgery.^(5)^

In this procedure, a neuromodulation system comprises three parts: quadripolar brain electrodes, a neurostimulator, and an extension cord. Electrodes are implanted using stereotaxic surgery guided by imaging tests and intraoperative physiological confirmation.^(6)^ Several factors must be evaluated to determine the candidacy of a patient with PD for DBS. First, the diagnosis is evaluated because the treatment is more effective and appropriate in patients with idiopathic PD. Second, it is necessary to verify which symptoms and signs are most problematic or disabling for the patient, confirm the ineffectiveness of pharmacological therapy, and determine whether these symptoms can be alleviated by DBS.^(7)^

Deep brain stimulation of the STN is effective in managing motor symptoms in advanced PD. The use of this treatment has increased and, as a result, has improved the quality of life of selected patients. However, some studies have reported possible cognitive, psychiatric, and behavioral changes caused by the procedure.^(4,8)^

The basal ganglia circuitry plays a central role in selecting and inhibiting movements, emotions, behaviors, and thoughts. Thus, DBS of the STN can alter non-motor functional networks and domains and, in turn, trigger psychiatric disorders. Nonetheless, no consensus has been reached in this regard because the neurodegeneration pattern of PD itself can cause these symptoms, making it difficult to determine the factors responsible for triggering them.^(9)^

## OBJECTIVE

To evaluate psychiatric alterations resulting from deep brain stimulation of the subthalamic nucleus in the management of Parkinson’s disease.

## METHODS

This systematic literature review was conducted in accordance with the recommendations of the Preferred Reporting Items for Systematic Reviews and Meta-Analyses (PRISMA).^(10)^

### Search strategy

The descriptors were chosen from the Health Sciences Descriptors/Medical Subject Headings (DeCS/MeSH) based on the three central themes of this research: patients with Parkinson’s disease, deep brain stimulation of the subthalamic nucleus, and psychiatric disorders (including mental disorders and social behavior disorders).

The articles were searched using three information databases: Public/Publisher MEDLINE (PubMed), Virtual Health Library (VHL), and Cochrane Library. The following descriptors indexed on the DeCS/MeSH database were associated with the Boolean operators “AND” and “OR.”

PubMed/Medline: (Parkinson Disease [MeSH Terms] OR Parkinson’s Disease [MeSH Terms]) AND (Deep Brain Stimulation [MeSH Terms] OR DBS [MeSH Terms] OR (Brain Stimulation [MeSH Terms] AND Subthalamic Nucleus [MeSH Terms])) AND (Mental Disorders [MeSH Terms] OR Social behavior disorders [MeSH Terms])=77 results. Virtual Health Library (VHL): (Parkinson Disease [MeSH Terms] OR Parkinson’s Disease [MeSH Terms]) AND (Deep Brain Stimulation [MeSH Terms] OR DBS [MeSH Terms] OR Brain Stimulation [MeSH Terms]=55 results. Cochrane Library: (Parkinson Disease [MeSH Terms] OR Parkinson’s Disease [MeSH Terms]) AND (Deep Brain Stimulation [MeSH Terms] OR DBS [MeSH Terms] OR Brain Stimulation [MeSH Terms]=13 results.

### Eligibility criteria

Fully indexed articles of randomized controlled trials, clinical trials, and case reports, written in English, Portuguese, or Spanish and published in the last 10 years relating to psychiatric alterations after DBS of the STN in patients with PD, were selected. No sex or age criteria were used for article eligibility. Articles that were incomplete and those that did not meet the objectives were excluded.

### Data collection process

Two researchers selected the search terms, filters, and databases that should be consulted and independently performed the search using the pre-established criteria. Subsequently, they independently reviewed the titles and abstracts of the 145 articles initially selected in the searches and discussed inconsistencies until they reached a consensus with a third researcher regarding the articles that should be selected for full-text evaluation.

## RESULTS

### Literature screening and evaluation

**Figure 1:**
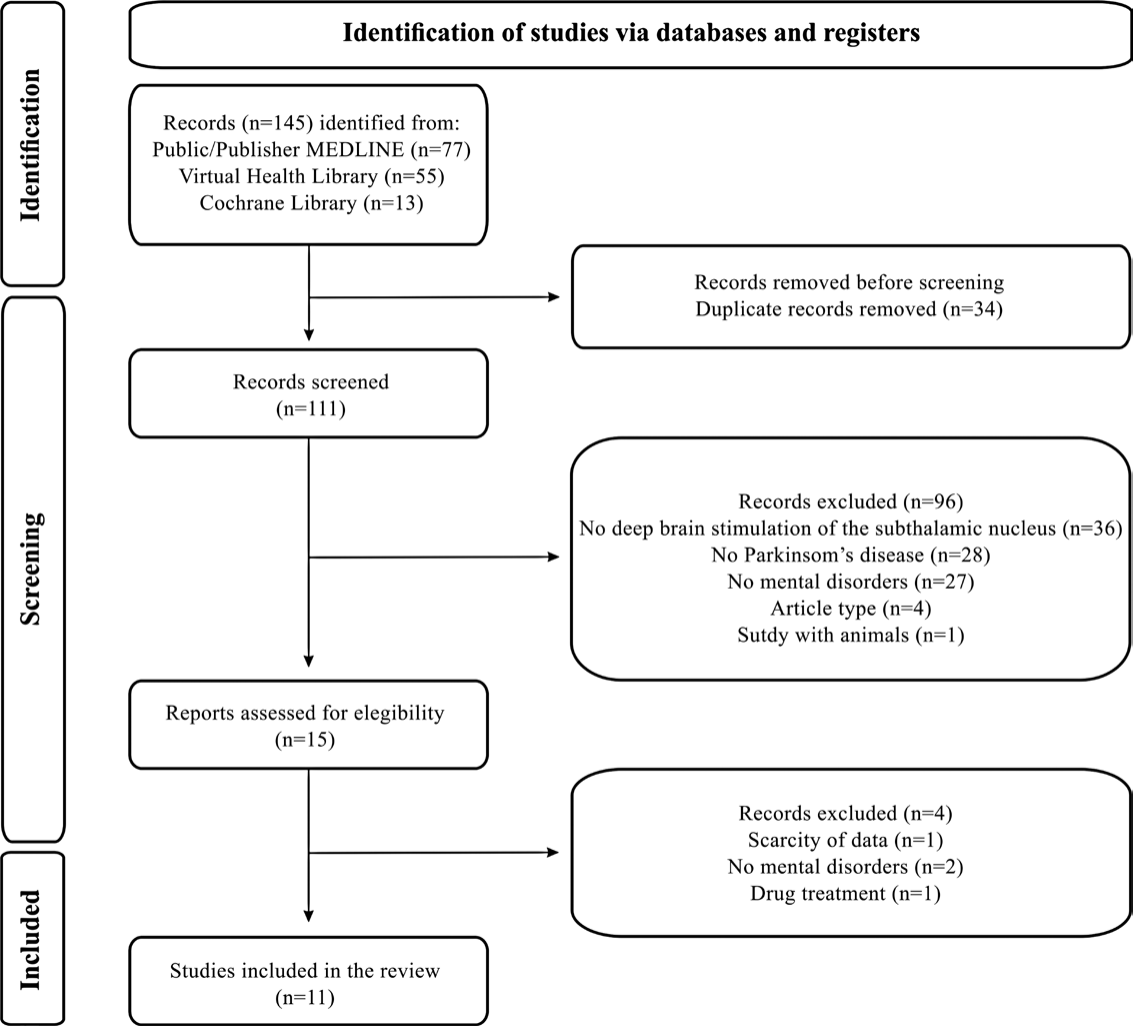
Identification of study

A total of 111 articles were identified during database analysis and after removing duplicates. After reading titles and abstracts, 96 articles were eliminated, and a full-text review of the 15 selected articles was performed. Of these, four clinical studies were excluded: one did not provide specific data on the sample submitted for DBS of the STN, two did not address psychiatric disorders, and one prioritized the pharmacological aspects of PD treatment. Thus, two randomized controlled trials, three clinical trials, and six case reports were included in the qualitative analysis, totaling 11 articles (Figure 1 and Table 1).^(11-21)^

**Table 1:** Characteristics of the articles selected in this systematic literature review

Reference	Study design	Sample and sex	Mean age (years)	Mean duration of PD (years)	DBS of STN	Psychiatric alteration	Etiology	Validated instrument for psychiatric evaluation
Zonana et al.^(11)^	Case report	1 male	51	10	Bilateral	Apathy, psychosis, depression, suicidal ideation	Microlesions near the medial forebrain bundle and electrode placement	NA
Mondillon et al.^(12)^	Randomized controlled trial	5 females 9 males	62.2	12.8	Bilateral	*Deficit* in the identification of negative emotional facial expressions	Orbitofrontal gyrus stimulation	NA
Schilbach et al.^(13)^	Case report	1 male	48	16	Bilateral (ventral border)	Hypomanic syndrome	Electrode positioned and stimulation of areas close to STN	NA
Okun et al.^(14)^	Clinical trial	3 females	58	12.1	Unilateral	Anxiety, depression, compulsion	Stimulation of ventral areas close to STN	Hamilton Depression Rating Scale, Hamilton Anxiety Rating Scale, Yale-Brown Obsessive-Compulsive Rating Scale, Apathy Scale, Young Mania Rating Scale, Beck Depression Inventory-II, Beck Anxiety Inventory
Ugurlu et al.^(15)^	Case reports	1 female 1 male	56.5	11	Bilateral	Manic syndrome, hypersexuality, psychosis	Previous picture of psychiatric disorder and stimulation of the ventral part of STN	Hamilton Depression Rating Scale, Young Mania Scoring Scale
Gee et al.^(16)^	Clinical trial	6 females 10 males	57.63	12.19	Bilateral	Impulsiveness	Stimulation of unintended areas of STN	Neuropsychological testing, Questionnaire for Impulsive-Compulsive Disorders in Parkinson’s Disease Rating Scale, Balloon Analogue Risk Task (some patients) and auditory prepulse inhibition testing (some patients)
Boel et al.^(17)^	Clinical trial	four	60.7	12	Bilateral	Depression	NA	Young Mania Rating Scale, Hospital Anxiety and Depression Scale
Scangos et al.^(18)^	Case report	1 male	50	9	Bilateral (ventral border)	Acute hypomania and residual mania	High-frequency stimulation of the ventromedial region of STN	Neuropsychiatric Inventory Questionnaire, Beck Depression Inventory
Gilbert et al.^(19)^	Case report	1 female	46	6	Bilateral	Mania, hypersexuality, depression, self-alienation	NA	NA
Lhommée et al.^(20)^	Randomized controlled trial	17	52	7.3	Bilateral	Psychosis, anxiety, depression, suicidal ideation	Stimulation of STN itself and underlying areas	Beck Depression Inventory II, Starkstein Apathy Scale, Ardouin Scale of Behavior in Parkinson’s Disease
Mosley et al.^(21)^	Case report	1 male	64	5	Bilateral	Manic syndrome (psychotic episodes)	Electrode positioned in the ventromedial area of STN	NA

PD: Parkinson’s disease; STN: subthalamic nucleus; DBS: deep brain stimulation.

Data extraction was performed by two researchers, independently, from 22 tables prepared using Microsoft^®^ Word, with the following variables of the studies: year and place of publication, country of origin, study design, number of centers and participants, funding sources, age and sex of participants, duration of illness, surgical intervention, electrode placement, psychiatric alterations, neuroanatomical notes, and validated instruments for evaluating the outcome and authors’ interests.

The data of two tables referring to the same study were compared, and the risks of bias were assessed with the help of a third researcher. No automated tools were used for this process.

### Study characteristics

In the 11 articles selected in this study, 61 patients with PD underwent DBS of the STN and had psychiatric disorders, of which 24 were male (39.3%), 16 were female (26.2%), and the sex of 21 patients could not be identified during data collection (34.4%). The mean age of this population was 56.83 (46–62) years, with a mean PD duration of 10.66 (5–16).

All patients underwent DBS of the STN; in 58 of them, the stimulation was bilateral (95%), whereas in three, it was unilateral (5%) except for one patient (1.7%) who had minimal bleeding adjacent to the left lateral ventricle and in the thalamus region.^(11)^ No other complications during surgery were reported.

### Psychiatric disorders

The validated instruments used in psychiatric evaluation studies are listed in table 1. Depressive disorder was the most frequent psychiatric disorder after DBS of the STN, which was cited in five of the 11 articles analyzed,^(11,14,17,19,20)^ encompassing 26 patients (42%) with a mean age of 53.76 (46–60) years and mean PD duration of 8.63 (6–12) years. In four of these articles, depression was associated with other psychiatric disorders, such as psychosis (n=18),^(11,20)^ suicidal ideation (n=18),^(11,20)^ hypersexuality (n=1),^(19)^ and anxiety (n=20).^(14,20)^ This psychiatric change occurred acutely soon after the procedure, and in one case report, no improvement was observed even after ceasing stimulation.^(11)^

In one clinical trial, slight increases in depression (Hamilton Depression Rating Scale) and anxiety (Hamilton Anxiety Rating Scale) were noted in female patients undergoing unilateral DBS of the STN.^(14)^ In addition, another study indicated that patients who were already diagnosed with depression before this treatment obtained relief from the symptoms of PD and a reduction in the use of medication.^(20)^

Manic syndrome alone was reported in two of the 11 studies.^(13,18)^ Psychosis alone was not reported in any of the studies analyzed, but it was found in association with other psychiatric alterations, not including manic syndrome.^(11,20)^ In two studies, an association between manic syndrome and psychotic episodes was observed.^(15,21)^ In the four studies that addressed psychosis, the sample included 21 patients, with a mean age of 52.95 (51–64) years and mean PD duration of 7.67 (5–11) years,^(11,15,20,21)^ whereas the five articles that mentioned manic/hypomanic syndrome included six patients, with a mean age of 54.6 (46–64) years and mean PD duration of 8.4 (5–16) years.^(13,15,18,19,21)^

The presentation of manic syndrome with psychotic episodes occurred acutely after DBS of the STN in three patients of the two studies.^(15,21)^In one of them, a female patient with a previous diagnosis of depression controlled with the use of citalopram (20mg/d) presented with manic syndrome and psychotic episodes 7 days after bilateral stimulation of the STN, requiring suspension of the medication and prescription of olanzapine (10mg/d), which was gradually reduced until the condition was completely controlled and there was no need to change the position or voltage of the electrode.^(15)^ In another case in the same study, a male patient, without any previous psychiatric alterations, presented an abrupt onset of mania with psychotic episodes 24 hours after changes in the DBS parameter of the STN, with a gradual decrease within 3 days after moving the electrodes to the more dorsal area of the STN.^(15)^ In a third case, a male patient had psychiatric alterations after the ventral electrode contact to treat residual motor symptoms. However, even after discontinuing DBS of the STN, the symptoms of mania with psychosis persisted for six weeks. The patient required hospitalization and psychiatric treatment with lithium (0.8mmol/L) and quetiapine (100mg/d). After controlling for this condition, DBS of the STN was gradually reintroduced until the symptoms were controlled without psychiatric involvement.^(21)^

Hypomanic syndrome was cited in two case reports, in which two male patients with PD underwent bilateral DBS of the STN, with electrodes positioned close to the ventral area of the nucleus, resulting in acute hypomania.^(13,18)^ In one report, stimulation of the dorsal region of the STN was no longer effective in achieving motor control two years after surgery, requiring the electrode to be repositioned to a more ventral area.^(13)^ In the other report, the reduction in the stimulation frequency from 130Hz to 5Hz resulted in significant control of hypomanic symptoms after 48 hours.^(18)^

Hypersexuality was associated with depression and self-alienation in another case report in which a female patient with PD underwent bilateral DBS of the STN.^(19)^ In a clinical trial that included 16 individuals with a mean age of 57.63 years and a mean PD duration of 12.19 years, bilateral DBS of the STN was effective in reducing impulse control disorders in 75% of the sample (12 patients).^(16)^

Finally, in one study, patients who underwent DBS of the STN showed *deficits* in identifying negative emotional facial expressions; this led to a decrease in glucose metabolism in the right orbitofrontal cortex.^(12)^

## DISCUSSION

### Neuroanatomical analysis of the subthalamic nucleus

#### Anatomical relationships

The STN is a small biconvex structure located at the diencephalic-mesencephalic junction. It is medially limited to the internal capsule, dorsally to the substantia nigra, and ventrally to the thalamus. Together with the subthalamic fasciculus and the lenticular loop, it forms the subthalamus.^(22)^

These neuroanatomical notes have pre-surgical relevance, as the propagation of electricity to structures adjacent to the STN during DBS has been identified as a probable etiology of the aforementioned psychiatric alterations.^(13,14,16)^ One of the studies analyzed in this review indicated that stimulation of the medial forebrain bundle is related to manic and depressive symptoms. Furthermore, stimulation of the left substantia nigra was strongly associated with the appearance of depressive symptoms.^(11)^

Other studies have reported that an increase in stimulation frequency, aimed at better control of motor symptoms, may be one of the factors associated with the propagation of electricity to structures proximal to the STN, such as the medial forebrain bundle^(15)^ and substantia nigra.^(13)^ Accordingly, in one case report, a reduction in DBS frequency from 130 Hz to 5 Hz was sufficient to dissipate a patient’s acute hypomania.^(18)^ This result raises the hypothesis that some psychiatric disorders following DBS of the STN are voltage-dependent.

#### Subthalamic nucleus tripartite functional theory

Neuroanatomical evidence suggests that the medial, ventromedial, and dorsolateral parts of the STN correspond to the limbic, associative, and motor subdivisions, respectively, making up the tripartite functional theory of the STN.^(23)^ Moreover, neuroimaging studies have shown projections from the anterior region of the STN to the basolateral nucleus of the amygdala, anterior hippocampus, posteromedial internal globus pallidus, middle external globus pallidus, and anterior thalamic nucleus. In contrast, the posterior region presented connections with the posterior third of the putamen and external globus pallidus, middle caudal region of the caudate nucleus, posterior end of the hippocampus, and ventrolateral nucleus of the thalamus.^(24)^

Corroborating these findings, several authors have hypothesized that stereotaxic insertion of the electrode close to the ventromedial surface of the STN, the region of passage of the limbic and associative pathways, would be associated with the psychiatric alterations previously described.^(11,13,15,16,18,21)^ This could be a neuroanatomical explanation for the etiology of the changes based on the tripartite theory of the STN.^(23)^

#### Microlesions and neuroplasticity

Another study hypothesized that psychiatric symptoms may also be related to microlesions caused by electrode implantation during surgical procedures. This assumption was based on the fact that one patient had an acute psychotic condition before the activation of the device, even with no alterations on cranial computed tomography, magnetic resonance imaging, electroencephalography, and carotid Doppler.^(11)^ In another patient, prolonged and uninterrupted DBS of the STN led to a manic syndrome that persisted even after the stimulus was inactivated. This finding is related to the possible neuroplasticity of non-motor circuits modulated by stimulation.^(21)^

#### Psychiatric disorders

The most frequent psychiatric disorders in the selected studies were depression (45.4%), manic/hypomanic syndrome (45.4%), psychosis (36.6%), anxiety (18.2%), suicidal ideation (18.2%), and hypersexuality (18.2%). In addition, apathy (9.1), *deficits* in identifying negative emotional facial expressions (9.1%), and impulsivity (9.1%) were cited less frequently.

#### Manic/hypomanic syndrome and psychosis

The manic/hypomanic syndrome associated with psychotic episodes induced by DBS of the STN occurred abruptly and perceptibly in one of the analyzed studies. It was mainly related to the placement of the electrode in the ventromedial region of the STN and the frequency of stimulation.^(15)^ Previous conditions or predispositions to psychiatric diseases are considered probable triggers for this syndrome.

The participants in the analyzed studies generally reported euphoria associated with symptoms of grandiosity, insomnia, racing thoughts, and increased speed of speech,^(13,15,21)^ as well as delusions and auditory hallucinations.^(11)^ Some engaged in risky activities, such as gambling and dangerous driving.^(11,21)^

Remediation of mania/hypomania secondary to DBS of the STN can be achieved by reducing the amplitude/frequency of stimulation or changing the active contact of the electrode to a more dorsal position.^(13,15,18,21)^ Furthermore, pharmacological treatment with mood stabilizers was deemed positive in one of the studies, opening the possibility of an optional treatment when the objective is to maintain the motor benefits provided by electrode placement or when this psychiatric change persists after DBS discontinuation.^(13)^

#### Depression, anxiety, and suicidal ideation

In the studies analyzed, depression was one of the most frequent psychiatric disorders after DBS of the STN in patients with PD. In most cases, its presentation was acute^(11,19,20)^ or associated with other disorders, such as anxiety, psychosis, and suicidal ideation.^(11,14,19,20)^

Depressive disorder is one of the non-motor symptoms most frequently found in patients with PD. Its etiopathogenesis is multifactorial, involving social and psychological factors, including movement oscillations and pathological alterations of the dopaminergic system caused by the disease.^(25,26)^The relationship between DBS of the STN and depression is complex. It may involve several factors, such as the predisposition of patients to this disorder,^(11)^ direct action of stimulation of the dopaminergic pathway,^(18)^ and inhibition of specific regions of the STN, namely the ventromedial area or adjacent structures such as the medial forebrain bundle and substantia nigra.^(11)^

In addition, in one study, the abrupt reduction of dopaminergic medication after DBS of the STN was considered one of the factors related to post-stimulation depression.^(14)^ This finding is in agreement with another study that reported that dopamine agonists can have an antidepressant effect in patients with PD.^(27)^ In contrast, some patients who had already been diagnosed with depression before undergoing DBS of the STN achieved relief from the symptoms of the disease and a reduction in the use of medication after the procedure.^(20)^ However, whether this was due to the direct limbic action of STN stimulation or the detriment of the improvement in motor symptoms that this surgery provided remains unknown because motor impairment is one of the main factors responsible for depression and decline in quality of life in patients with PD.^(28)^

#### Impulsiveness

In one of the selected articles, DBS of the STN improved impulsiveness in a patient with PD who was diagnosed with this disorder before the surgical procedure.^(16)^ In contrast, a recent study demonstrated a relationship between increased impulsivity and DBS of the STN.^(29)^ The possible explanation is that DBS would release the “brake” of STN, leading to rapid responses, especially in conflict situations. This suggests a diminished ability to maintain initial response tendencies under control and raises the hypothesis of alterations in the mesolimbic reward system, as dopaminergic neurons facilitate behavior adaptation.

#### Research limitations

Finally, the study was limited by a small sample size owing to the low number of publications on the topic. Other limitations were imposed by the difficulty of the selected studies in standardizing the instruments for assessing mood and detailing the surgical technique and stimulated anatomical targets more precisely.

## CONCLUSION

Based on our findings, deep brain stimulation of the subthalamic nucleus for patients with Parkinson’s disease is a neurosurgical treatment already available and used. However, the psychiatric manifestations after implantation and activation remain poorly understood. The occurrence of more common psychiatric disorders related to the neuroanatomy of the nucleus was observed, probably because of the microlesions caused by the implantation of deep brain stimulation and regulation of the stimulation of the device. The most common disorders include depression, mania/hypomania, psychosis, anxiety, suicidal ideation, and hypersexuality.

Some previous conditions related to a predisposition to these disorders have been mentioned by some authors, such as low dopaminergic response to pharmacological treatments, advanced age, and underlying psychiatric illnesses. These factors have been identified as conditions that can lead to psychiatric manifestations after deep brain stimulation of the subthalamic nucleus. In contrast, certain studies have provided data related to impulsivity, which raises the hypothesis that implementing deep brain stimulation brings secondary benefits to patients in terms of impulsivity control, in addition to the already advocated motor control of Parkinson’s disease.

Therefore, deep brain stimulation of the subthalamic nucleus for patients with Parkinson’s disease is a complex technique under development and optimization, mainly when it comes to its potentially debilitating psychiatric symptoms. Consequently, studies with greater scientific evidence on patient selectivity, functional neuroanatomy of the subthalamic nucleus related to psychiatric symptoms, and the treatment of these stimulus-dependent disorders are necessary for a sensitive and specific assessment of the proposed topic.

## References

[1] Cabreira V, Massano J (2019). Doença de Parkinson: revisão clínica e atualização. Acta Med Port.

[2] Kaasinen V, Vahlberg T, Suominen S (2015). Increasing age-adjusted male-to-female incidence ratio of Parkinson's disease. Mov Disord.

[3] Martins CC, Caon G, Moraes CM (2020). A doença de Parkinson e o processo de envelhecimento motor: uma revisão de literatura. Saude Des Hum.

[4] Hariz MI, Blomstedt P, Zrinzo L (2010). Deep brain stimulation between 1947 and 1987: the untold story. Neurosurg Focus.

[5] Hamani C, Florence G, Heinsen H, Plantinga BR, Temel Y, Uludag K (2017). Subthalamic Nucleus Deep Brain Stimulation: Basic Concepts and Novel Perspectives. eNeuro.

[6] Chiken S, Nambu A (2016). Mechanism of deep brain stimulation: inhibition, excitation, or disruption?. Neuroscientist.

[7] Marks WJ (2010). Deep brain stimulation management.

[8] Dayal V, Limousin P, Foltynie T (2017). Subthalamic nucleus deep brain stimulation in Parkinson's disease: the effect of varying stimulation parameters. J Parkinsons Dis.

[9] Volkmann J, Daniels C, Witt K (2010). Neuropsychiatric effects of subthalamic neurostimulation in Parkinson disease. Nat Rev Neurol.

[10] Page MJ, McKenzie JE, Bossuyt PM, Boutron I, Hoffmann TC, Mulrow CD (2021). The PRISMA 2020 statement: an updated guideline for reporting systematic reviews. BMJ.

[11] Zonana J, Zimmerman M, McCarty SS, Ferrando S (2011). A case of abrupt-onset apathy, psychosis, and depression following deep brain stimulation in a patient with Parkinson's disease. Psychosomatics.

[12] Mondillon L, Mermillod M, Musca SC, Rieu I, Vidal T, Chambres P (2012). The combined effect of subthalamic nuclei deep brain stimulation and L-dopa increases emotion recognition in Parkinson's disease. Neuropsychologia.

[13] Schilbach L, Weiss PH, Kuhn J, Timmermann L, Klosterkötter J, Huff W (2012). Pharmacological treatment of deep brain stimulation-induced hypomania leads to clinical remission while preserving motor benefits. Neurocase.

[14] Okun MS, Wu SS, Fayad S, Ward H, Bowers D, Rosado C (2014). Acute and chronic mood and apathy outcomes from a randomized study of unilateral STN and GPi DBS. PLoS One.

[15] Ugurlu TT, Acar G, Karadag F, Acar F (2014). Manic episode following deep brain stimulation of the subthalamic nucleus for Parkinson's disease: a case report. Turk Neurosurg.

[16] Gee L, Smith H, De La Cruz P, Campbell J, Fama C, Haller J (2015). The influence of bilateral subthalamic nucleus deep brain stimulation on impulsivity and prepulse inhibition in Parkinson's disease patients. Stereotact Funct Neurosurg.

[17] Boel JA, Odekerken VJ, Schmand BA, Geurtsen GJ, Cath DC, Figee M, van den Munckhof P, de Haan RJ, Schuurman PR, de Bie RM, NSTAPS study group (2016). Cognitive and psychiatric outcome 3 years after globus pallidus pars interna or subthalamic nucleus deep brain stimulation for Parkinson's disease. Parkinsonism Relat Disord.

[18] Scangos KW, Shahlaie K (2017). Acute frequency-dependent hypomania induced by ventral subthalamic nucleus deep brain stimulation in Parkinson's disease: a case report. Biol Psychiatry.

[19] Gilbert F, Viaña JN (2018). A personal narrative on living and dealing with psychiatric symptoms after DBS surgery. Narrat Inq Bioeth.

[20] Lhommée E, Wojtecki L, Czernecki V, Witt K, Maier F, Tonder L, Timmermann L, Hälbig TD, Pineau F, Durif F, Witjas T, Pinsker M, Mehdorn M, Sixel-Döring F, Kupsch A, Krüger R, Elben S, Chabardès S, Thobois S, Brefel-Courbon C, Ory-Magne F, Regis JM, Maltête D, Sauvaget A, Rau J, Schnitzler A, Schüpbach M, Schade-Brittinger C, Deuschl G, Houeto JL, Krack P, EARLYSTIM study group (2018). Behavioural outcomes of subthalamic stimulation and medical therapy versus medical therapy alone for Parkinson's disease with early motor complications (EARLYSTIM trial): secondary analysis of an open-label randomised trial. Lancet Neurol.

[21] Mosley PE, Marsh R, Perry A, Coyne T, Silburn P (2018). Persistence of mania after cessation of stimulation following subthalamic deep brain stimulation. J Neuropsychiatry Clin Neurosci.

[22] Mavridis I, Boviatsis E, Anagnostopoulou S (2013). Anatomy of the human subthalamic nucleus: a combined morphometric study. Anat Res Int.

[23] Karachi C, Yelnik J, Tandé D, Tremblay L, Hirsch EC, François C (2005). The pallidosubthalamic projection: an anatomical substrate for nonmotor functions of the subthalamic nucleus in primates. Mov Disord.

[24] Lambert C, Zrinzo L, Nagy Z, Lutti A, Hariz M, Foltynie T (2012). Confirmation of functional zones within the human subthalamic nucleus: patterns of connectivity and sub-parcellation using diffusion weighted imaging. Neuroimage.

[25] Schrag A (2006). Quality of life and depression in Parkinson's disease. J Neurol Sci.

[26] Postuma RB, Berg D, Stern M, Poewe W, Olanow CW, Oertel W (2015). MDS clinical diagnostic criteria for Parkinson's disease. Mov Disord.

[27] Barone P, Poewe W, Albrecht S, Debieuvre C, Massey D, Rascol O (2010). Pramipexole for the treatment of depressive symptoms in patients with Parkinson's disease: a randomised, double-blind, placebo-controlled trial. Lancet Neurol.

[28] Schrag A, Jahanshahi M, Quinn N (2000). What contributes to quality of life in patients with Parkinson's disease?. J Neurol Neurosurg Psychiatry.

[29] Lo Buono V, Lucà Trombetta M, Palmeri R, Bonanno L, Cartella E, Di Lorenzo G (2021). Subthalamic nucleus deep brain stimulation and impulsivity in Parkinson's disease: a descriptive review. Acta Neurol Belg.

